# Molecular mechanism of *Cinnamomum zeylanicum* and *Citrus aurantium* essential oils against the root-knot nematode, *Meloidogyne incognita*

**DOI:** 10.1038/s41598-025-90529-8

**Published:** 2025-02-19

**Authors:** Elena Fanelli, Alessio Vovlas, Trifone D’Addabbo, Francesca De Luca

**Affiliations:** Institute for Sustainable Plant Protection-CNR, Via Amendola 122/D, Bari, 70126 Italy

**Keywords:** Essential oils, Gene expression, *Mi-ace*-1, *Mi-ace*-2, *Mi-far*-1, *Mi-hsp*90, Root-knot nematode, Toxicity mechanism, Nematode control, Molecular biology, Plant sciences

## Abstract

The root-knot nematode *Meloidogyne incognita* is the most harmful and destructive nematode species due to its widespread distribution and its ability to destroy a wide range of agriculturally and economically important crops. Plant-derived essential oils (EO) are one of the promising strategies for nematode management showing high nematicidal potential. Very little is known about the molecular mechanisms activated by EOs in *M. incognita*. In our study, we assessed the effects of *Cinnamomum zeylanicum*,* Citrus aurantium* and the chemical nematicide Oxamyl on the mortality of *M. incognita* juveniles (J2) at different doses and exposure. The toxicity of *C. zeylanicum* and *C. aurantium* EOs to *M. incognita* J2 was largely different, peaking 94.7 and 26.7% mortality rates after a 24-hour exposure to 100 µg mL^− 1^ EO concentrations, respectively. The different nematicidal activity of the two EOs was also confirmed by their LC_50_ values, ranging between 0.1 and 399 mg mL^− 1^ concentrations for the 24-hour treatment, respectively.

The sublethal dose (0.78 µg mL^− 1^), the concentration that does not cause mortality but still triggers physiological responses, was chosen for both EOs and Oxamyl to investigate the expression levels of *Mi-ace*-1, *Mi-ace*-2, and *Mi-hsp*90, involved in host finding processes, and *Mi-far*-1, involved in the cuticle protection, by using real time PCR. Our results reveal that *C. zeylanicum* is toxic at lower doses than *C. aurantium*. The different expression level of *Mi-far*-1 in J2s treated with *C. zeylanicum* suggests a different action mode compared to *C. aurantium* and Oxamyl. Understanding the action mode of these compounds at molecular level will help to develop more potent and effective nematicides safer for environment and humans.

**Supplementary Information:**

The online version contains supplementary material available at 10.1038/s41598-025-90529-8.

## Introduction

Plant parasitic nematodes are included among the most damaging crop pests, as globally causing yield losses of up to USD 157 billion per year^1,2^. Root-knot nematodes (*Meloidogyne* spp.) are considered the most harmful, as worldwide spread on a wide range of herbaceous and tree crops and can cause complete yield loss of crop yield^3,4^. In the past century, control of RKN infestations has been primarily based on soil treatments with chemical nematicides, highly toxic to human health and environment, prompting the search for new effective, but safer nematicidal products^5^. Biological strategies, as use of beneficial fungi, bacteria, plants and essential oils, have proven to be good alternatives for nematode management. In this effort, plant secondary metabolites with biocidal properties, also including essential oils (EOs) have been widely investigated in the last two decades^6,7^.

EOs are mixtures of volatile substances with different chemical composition, as terpenoids, aromatic phenols, oxides, ethers, alcohols esters, aldehydes, and ketones, present in a large variety of aromatic plants^8^. Using EOs or their components in commercial pesticides formulation is getting interest both in the scientific and industrial fields, as well as decreasing environmental impact^9^. The nematicidal activity of these products has been widely demonstrated to be linked to their chemical structures, as each individual component is responsible of the mode of action of EOs^10–12^. Due to the lipophilic and low molecular structures, most of EO components affect nematodes using different mechanisms such as disrupting their nervous systems, plasma membrane permeability, or penetrating the gelatinous matrix of eggs^13,14^.

The nervous system of RNKs facilitate nematodes movement and infection process using their chemoreceptive neurons to sense distinct chemical stimuli for the invasion of host plants^15^. Nematodes use acetylcholinesterase (*AChE*) and/or nicotinic acetylcholine receptors for synaptic transmission and locomotion processes. *AChE* is also a well-known and studied pesticide target site in nematodes^16,17^. Three different genes for acetylcholinesterase, *Mi*-*ace*-1, *Mi*-*ace*-2, and *Mi*-*ace*-3 were found in *M. incognita*^18–20^.

Other target sites of Eos are the cuticle and the amphids, structures in permanent contact with the environment, producing secretory proteins. At amphids level is localized Hsp90, a family containing highly conserved genes, mainly involved in chaperone and folding activities during abiotic stress (environment and chemicals) as well as in chemoreception toward host plants^21–24^.

Cuticle proteins from PPNs interact with the environment and host plants playing an important role in parasitism^25^. Among these, *Mi*-*far*-1 protein, localized in the hypodermis of *M. incognita*, is of particular interest as playing a dual role: being involved in the binding of host fatty acids in addition to retinols and participating in the modulation of host susceptibility to PPNs^26–29^. Limited studies are available about how nematode gene expression is altered in response to EOs exposure, although more information is needed to understand their nematicidal effects in order to develop new potential nematicides^7,30^.

The present study aimed to delucidate and to compare the molecular action mechanisms of two EOs, *C. zeylanicum* and *C. aurantium*, and the chemical nematicide Oxamyl on *M. incognita* juveniles (J2) by following the expression levels of four different genes involved in motility, chemoreception and protection mechanisms. The results clearly demonstrated that *C. zeylanicum* is much toxic compared to *C. aurantium* and Oxamyl. The molecular action mode for *C. zeylanicum* is the disruption of the protective action of *Mi*-*far*-1 in the cuticle.

## Results

### Toxicity to *M. incognita* J2

The mortality of *M. incognita* J2s was negligible in sterile water and 0.3% Tween-20 solution. Mortality rates of *M. incognita* J2 treated with the *C. zeylanicum* EO increased with EO concentration (Table 1), reaching 62% and 80% after 4-hour exposure to 6.25 and 12.5 µg mL^− 1^ concentrations, respectively. Mortality of *M. incognita* J2 treated with *C. aurantium* EO was largely lower than that caused by *C. zeylanicum* EO at all the tested concentrations x exposure time combinations (Table 1). The *C. aurantium* EO resulted in a peak of 26.7% J2 mortality after a 24-hour treatment with 100 µg mL^− 1^, while a similar mortality rate (25.8%) occurred at the 4 h exposure to 1.56 µg mL^− 1^ concentration of *C. zeylanicum* EO. The different nematicidal activity of the two EOs was clearly indicated also by the values of LC50, as corresponding to only 5.0, 1.67 and 0.1 µg mL^− 1^ at 4-, 8- and 24-hours, respectively, for the *C. zeylanicum* EO, while the *C. aurantium* EO LC50 values were about 1200 µg mL^− 1^ at 4- and 8-hours exposures and about 400 µg mL^− 1^ at 24-hours (Table 2). When compared to the positive control, Oxamyl, the mortality of *M. incognita* J2 was like that of *C. zeylanicum* EO at 100 µg mL^− 1^ × 24-hour combination (*p* ≤ 0.05). In addition, compared to Oxamyl, effect of *C. zeylanicum* on the mortality of J2 was observed early in the time course exposures, with clear differences recorded at 4- and 8-hours, at most concentrations tested (*p* ≤ 0.05).

**Table 1:** Percentage mortality (mean of four replicates ± SE)) of the infective juveniles of the root-knot nematode *Meloidogyne incognita* exposed for 4, 8–24 h to different concentrations of the EOs from *Cinnamomum zeylanicum* and *Citrus aurantium*.

Concentrations (µg m^L−1^)	Exposure time (hours)					
	4		8		24	
	*C. zeylanicum*	*C. aurantium*	*C. zeylanicum*	*C. aurantium*	*C. zeylanicum*	*C. aurantium*
0.78	15.2 ± 0.3	0.6 ± 0.1	36.1 ± 0.5	0.9 ± 0.1	64.5 ± 0.5	0.9 ± 0.1
1.56	25.8 ± 0.4	0.9 ± 0.1	41.3 ± 0.4	1.0 ± 0.1	71.8 ± 0.5	1.0 ± 0.1
3.12	41.5 ± 0.5	1.4 ± 0.1	63.7 ± 0.5	2.2 ± 0.1	81.2 ± 0.6	2.5 ± 0.1
6.25	62.4 ± 0.5	1.7 ± 0.1	72.3 ± 0.6	5.5 ± 0.4	89.5 ± 0.8	7.5 ± 0.7
12.5	79.5 ± 0.8	5.9 ± 0.4	83.3 ± 0.3	6.8 ± 0.3	90.4 ± 0.7	10.8 ± 0.5
25	79.7 ± 0.9	7.9 ± 0.5	85.3 ± 0.3	8.3 ± 0.4	91.0 ± 0.7	14.0 ± 0.4
50	83.4 ± 0.9	9.1 ± 0.8	89.2 ± 0.4	10.5 ± 0.7	91.2 ± 0.8	15.6 ± 0.6
100	88.5 ± 0.7	16.7 ± 0.7	89.5 ± 0.8	20.3 ± 0.9	94.7 ± 0.5	26.7 ± 0.9
Oxamyl	4.1 ± 0.6	4.1 ± 0.6	43.6 ± 0.9	43.6 ± 0.9	94.9 ± 0.9	94.9 ± 0.9
Tween 20	0	0	0.2 ± 0.1	0.2 ± 0.1	0.6 ± 0.1	0.6 ± 0.1
Water	0	0	0.1 ± 0.1	0.1 ± 0.1	0.5 ± 0.1	0.5 ± 00.1
LSD (0.05)	1.7	1.2	1.5	1.4	1.8	1.5

**Table 2:** The LC50 probit analysis parameters of the two tested essential oils from *Cinnamomum zeylanicum* and *Citrus aurantium* at 4-, 8- and 24-hour exposures of the *Meloidogyne incognita* J2.

Probit analysis	*C. zeylanicum* EO			*C. aurantium* EO		
	4 h	8 h	24 h	4 h	8 h	24 h
LC_50_	5.0	1.7	0.1	1200.1	1222.6	398.9
95% CI	2.6–9.7	0.7–3.9	0.02–0.4	325.8–4420.1	328.9–4544.5	144.1–1103.9
SE	0.144	0.190	0.321	0.289	0.291	0.226
R^2^	0.937	0.933	0.895	0.973	0.957	0.957

### Effects of essential oils on the expression of target genes

**Fig. 1:**
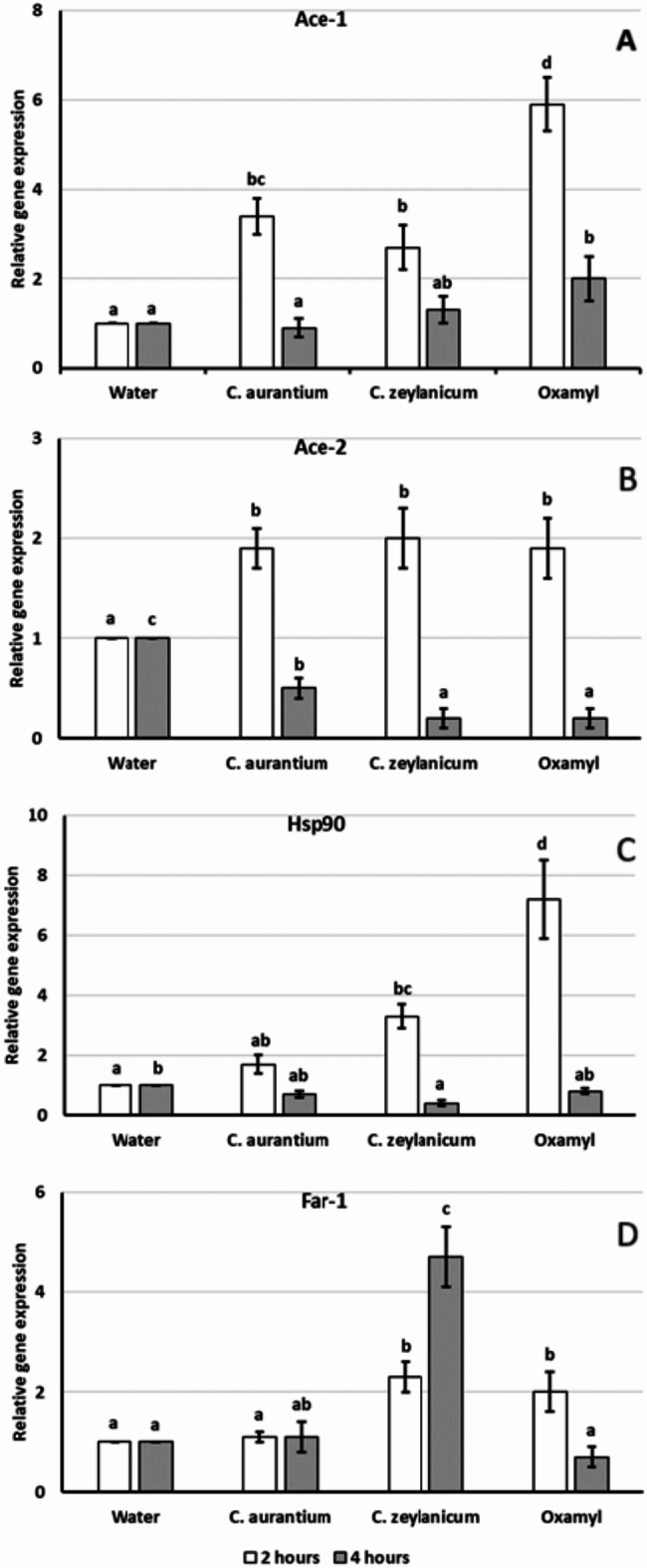
Relative expression of genes *Mi-ace*-1 (**A**), *Mi-ace*-2 (**B**), *Mi-hsp*90 (**C**) and *Mi-far*-1 (**D**) in *Meloidogyne incognita* after a 2- and 4-hour exposure to 0. 78 µg mL^− 1^ solutions of *Citrus aurantium*, *Cinnamomum zeylanicum* essential oils and to a 2 ml L^− 1^ water solution of Oxamyl. At each exposure time, bars marked with the same letters are not significantly different according to the Least Significant Difference Test (*P* ≤ 0.05).

The toxic effects of *C. zeylanicum* and *C. aurantium* Eos on the target genes of *M. incognita* J2s were evaluated after 2- and 4-hour exposure at 0.78 µg mL^− 1^ sublethal EO concentration, at which the nematode locomotion behavior was not yet disrupted and only few specimens were killed by *C. zeylanicum* EO. As changes in gene expression can be exploited to diagnose the effects of exposure to environmental chemicals, we investigated the expression of four different genes involved in the motility, chemoreception and cuticle protection mechanisms by qPCR, *Mi*-*ace*-1 and *Mi*-*ace*-2 (motility of the nematode), *Mi*-*hsp*90 (response to biotic and abiotic stress) *Mi*-*far*-1 (modulation of host susceptibility) genes. A fragment of 186 bp of 18S rRNA gene was used as endogenous control for normalization. The expression level of Mi-*hsp*90 increased by 3.3 and 7.2 folds (*p* < 0,01) after 2-hour incubation in nematodes treated with *C. zeylanicum* and Oxamyl, respectively, while with *C. aurantium* EO the expression level changed slightly (1.7 folds). After 4-hour exposure to both Eos and Oxamyl, *Mi-hsp90* expression decreased to basal level (Fig. 1C).

The relative expression of *Mi*-a*ce*-1 in J2s incubated with *C. aurantium*, *C. zeylanicum* and Oxamyl, after 2-hour treatment, increased (*P* < 0.01) by 2.7, 3.4 and 5.9 folds, respectively, and decreased at basal levels after 4-hour treatment, but in Oxamyl treated J2s the level was still higher than the untreated control (Fig. 1A, B). The relative expression of *Mi*-*ace*-2 in J2s incubated with *C. aurantium*, *C. zeylanicum* and Oxamyl increased about 2 folds after 2-hour treatment, but decreased (*P* < 001) by 50%, 75% and 80%, respectively, after 4-hour incubation compared to untreated control. *Mi*-*ace*-1 and *Mi*-*ace*-2 both respond to EO treatment showing the same expression pattern of Oxamyl, suggesting an identical impact on the nervous system. No significant variation of *Mi-fa*r-1 expression was observed in *C. aurantium* and Oxamyl treatments after 2- and 4- hour incubation, whereas *Mi*-*far*-1 relative expression increased by 2.3 and 4.7 folds in J2s treated with *C. zeylanicum* after 2 and 4 h treatment, respectively (Fig. D).

## Discussion

Plant EOs represent a potential control tool for insects and plant parasitic nematodes of economic importance and for the reduction of toxicity on non-target invertebrates^31,32^. This study demonstrated for the first time how *M. incognita* nematode gene expression level is altered after exposure to EOs, as contributing to elucidate the EOs mode of action.

In the current investigation, we report on the nematicidal activity of *C. zeylanicum* and *C. aurantium* EOs on *M. incognita* as previously demonstrated by D’Addabbo et al.^33^ confirming that *C. zeylanicum* recorded higher J2 mortality rate at lower doses and shorter incubation time compared to *C. aurantium*. Adversely, the treatment with Oxamyl achieved the same J2 mortality rate 94.9% of *C. zeylanicum* EO after the 24-hour exposure (Table 1). The different nematicidal activity of the two EOs was also confirmed by their LC_50_ values, ranging between 0.1 and 399 mg mL^− 1^ concentrations for the 24-hour treatment, respectively (Table 2). Our result confirms that *C. zeylanicum* EO could be a good strategy for *M. incognita* management, but more studies are needed to evaluate their efficacy in field. The mechanism of action on the target nematode has also gained attention in order to explore the possibility to develop resistance or adaptation to treatments.

Information on the mode of action of EOs and their constituents is of practical importance for nematode control, as providing useful indications on their most appropriate formulation and delivery means as well as on their environmental safety^12,33–37^. According to our previous chemical analysis of the same two EOs used in this study, the *C. zeylanicum* EO was prevalently made of phenolics such as E-cinnamaldehyde (84.8%) and eugenol (13.4%), while the dominant component of the *C. aurantium* EO was limonene (94.9% of the total EO), with small amounts of β-myrcene (1.6%), linalool (1.0%) and linalyl acetate (1.5%)^33^. Studies on insects revealed that several EOs and their components can penetrate the cuticle and act on internal lipids or AchE activity^38,39^. Furthermore, in the model nematode, *Caenorhabditis elegans*, has been shown that several essential oils can produce rapid paralysis^7,14,40,41^. In this context, we investigated the changes in gene expression of the stress related *Mi-hsp*90 gene, the neurotoxicity indicator *Mi*-*ace*-1 and *Mi*-*ace*-2 genes, and the cuticle integrity *Mi-far*-1 gene, upon sublethal exposure for both EOs and Oxamyl chemical. *Mi-hsp*90 expression responds promptly to 2 h treatment with *C. zeylanicum* and Oxamyl, resulting much higher in J2s treated with Oxamyl, thus indicating a stressful condition for J2s (Fig. 1C). No change of *Mi-hsp*90 expression was detected in J2 exposed to *C. aurantium*. After 4-hour incubation, *Mi-hsp*90 expression is restored to basal levels in all tested compounds suggesting nematode adaptation to stress condition. The expression levels of both neurotoxic *Mi-ace*-1 and *Mi-ace*-2 genes promptly increased after 2-hour treatments with both EOs and Oxamyl confirming the involvement of nervous system (Fig. 1B, C). After prolonged incubations, *Mi-ace*-1 expression levels were restored at basal level for both EOs, while in J2s treated with Oxamyl the expression was still high (Fig. 1A). *Mi-ace*-2 expression levels decreased below the control level with both EOs and Oxamyl (Fig. 1B). *Mi-far*-1 expression level increased in J2s exposed to *C. zeylanicum* after 2- and 4-hours suggesting the active role of the cuticle to contrast the higher toxic effects of *C. zeylanicum* compared with *C. aurantium* and Oxamyl (Fig. 1D). The different expression level of *Mi-far*-1 furtherly suggested that *C. zeylanicum* has a different action mechanism compared to *C. aurantium* and Oxamyl correlated to the different chemical compositions. These results demonstrated that both EOs and Oxamyl display toxic and stress effects on J2s, but the higher expression level of *Mi-far*-1 in J2s treated with *C. zeylanicum* confirms different action mode able to overcome the protective coat of *Mi-far*-1 and to penetrate through the cuticle causing the disruption of the nervous system and the cell membranes.

In conclusion, in the last decades different EOs for nematode management were tested in vitro, but most of them showed different effectiveness and were not tested in field conditions. Our study confirms the different early responses of *M. incognita* and the active role of the cuticle in contrasting the interaction with the EOs tested. Molecular and phenotypical knowledge of nematodes is very useful for developing new control strategies as plant parasitic nematodes are able to evolve and adapt to new biotic and abiotic conditions. It seems that *C. zeylanicum* has a potential to be included in new formulations, though its activity has to be validated in soil. In conclusion, the elucidation of the action mechanisms of EOs is a key factor for the formulation and field exploitation of new effective and safe EO-based nematicidal products.

## Methods

### Nematode Collection

Egg masses of *M. incognita*, previously reared on tomato cv. Roma in a glasshouse at 25°±2 C for two months, were handpicked from infested roots and then incubated in distilled water in a growth chamber at 25 °C. The emerged J_2_ were collected and stored at 5 °C until used in the experiments (one week) ensuring nematode viability and behavior. We extracted total DNA from individual *M. incognita* juveniles following the protocol outlined by De Luca et al.^42^. Nematodes were identified by ITS sequencing and amplifying specific region by SCAR-based PCR assay^43^.

## Nematode toxicity bioassay

Batches of 100 *M. incognita* J2 infective specimens were suspended in 0.5 ml distilled water and placed in 1.5 ml Eppendorf tubes. Appropriate amounts of commercial *C. zeylanicum* and *C. aurantium* EOs (Erboris Orientis Dacor, Milan, Italy) were added to a 0.3% Tween-20 water solution, as to prepare 1.56, 3.12, 6.25, 12.5, 25, 50, 100, 200 µg mL^− 1^ solutions. Tween-20 is a nonionic detergent used for the preparation of stable oil-in- water solutions and does not interfere with the activity of nematodes. A 0.5 ml volume of each solution was poured into each Eppendorf tube containing the nematode suspensions, as to obtain 0.78, 1.56, 3.12, 6.25, 12.5, 25, 50, 100 µg mL^− 1^ final test concentrations. The nematodes were exposed to each concentration for 4, 8, or 24 h. Distilled water, a 2 ml L^− 1^ water solution of a commercial formulation (Vydate^®^ 10 L, Corteva Agriscience, Cernay, France) the nematicide Oxamyl (10% a.i.) and 0.3% Tween-20 served as positive controls. Four replicates of each EO concentration x exposure time and of controls were provided. After each exposure time, J2 from each replicate were observed under a light microscope and ranked as motile or paralyzed by pricking the J2 body with a needle and assessments were made. After the microscopical observation as above, the nematodes were recovered on a 5-µm sieve and then transferred to distilled water. Nematode mortality was confirmed by the persistence of immobility at 72 h after placement in distilled water. Mortality rates were calculated according to the Abbott’s formula^44^ m = 100 × (1 − nt/nc), in which: m, percent mortality; nt, number of viable nematodes after the treatment; nc, number of viable nematodes in the water control. The 0.78 µg mL^− 1^ final test concentration was considered sublethal dose because complete viability was observed compared with the control. Two biological replicates were carried out for each treatment. For each biological replicate, three technical replicates were run. Data from the two experimental runs were pooled in the absence of a significant interaction of experiment x treatment^44^.

### Molecular characterization bioassay

Batches of 500 *M. incognita* J2 specimens suspended in 0.5 mL distilled water were placed in 1.5 mL Eppendorf tubes. Appropriate amount of *C. zeylanicum* and *C. aurantium* EOs were added to a 0.3% Tween-20 water solution.A 0.78 µg mL^− 1^ solution of each EO was added to the nematode suspensions.

A and incubated for 2 and 4 h as nematode locomotion and behavior were not disrupted. Three replicates were provided for each test solution × exposure time combination, including distilled water, a 2ml L^− 1^ water solution of the same commercial formulation Oxamyl, and a 0.3% solution of Tween-20 as controls. At the end of each experiment, nematodes were washed 3 times with distilled water and resuspended in a final volume of 30 µl distilled water. Samples were frozen in liquid nitrogen and stored at -80 °C until used.

## RNA extraction and target gene expression

Total RNA was extracted from 500 frozen J2, grounded in liquid nitrogen using a pestle and mortar. Total RNA was extracted, using a RNeasy mini kit (QIAGEN, Hilden, Germany), according to the manufacturer’s instructions. Residual genomic DNA was removed by incubation with 20 U of RNase-free DNase I (QIAGEN) for 15 min at room temperature. Total RNA concentration of each sample was measured by using NanoDrop (Thermofisher). Primers, used in the current study, were designed on *M. incognita* coding sequences present in GenBank: JQ683672 (*Mi-ace*-1), JQ701796 (*Mi-ace*-2), AF459026 (*Mi-hsp*-90), KF030975 (*Mi-far*-1), KU578066 (18S rRNA) by the authors according to standard PCR guidelines. For cDNA synthesis, 100 ng of total RNA was reverse transcribed in a 20 µl reaction mixture using a QuantiTect Reverse transcription kit (QIAGEN) following the manufacturer’s instructions. The relative expression among the life stages was calculated by using the ∆∆Ct method^45,46^. A portion of 18S rRNA gene was used as the endogenous control using specific primers: Q18SforMi (GCACCACCAGGAGTGGAG) and Q18SrevMi (TAGGTTAGAGTCTCGCTCG) Real-time PCR was performed in 20 µL volumes containing 10 ng of cDNA, 10 µl 2 × Fast Start SYBR Green master mix (Roche Diagnostics, GmbH, Mannheim, Germany) and 8 pmol of each specific primer. Gene-specific primers Mi-Ace1F (CCATCGCGCATCTCAACAA) and Mi-Ace1R (GCGGTTTGGGTCACCTGTA), MiAce2F(TGGATTTTACTCGGGCTCTC) and Mi-Ace2R (GGGCAATATGCTCATGTATCCAT), MiHsp90F (GACACGAAACCCCGATGACA) MiHsp90R (TGAACACACGTCGAACATAAAGC), Mi-Far1 (GATTTGGTCCCGCCTGAGG) and Mi-Far1R (TTGAATAACGCCGGTAATCTTGG) were used to determine the expression profile. Pilot experiments for real-time PCR were carried out to determine the right thermal profile for all gene targets. The thermal profile for real-time PCR was 10 min at 95 ^◦^C, followed by 40 cycles at 95 ^◦^C for 30 s, 58 ^◦^C for 30 s and 72 ^◦^C for 40 s. Dissociation curve analysis of the amplification products was performed at the end of each PCR to confirm that only one PCR product was amplified and detected. The real-time experiments were conducted on a MX3000P Q-PCR System (Stratagene, La Jolla, CA, USA), and fluorescent real-time PCR data were analyzed with MXpro Q-PCR Software (Stratagene).

### Statistical analysis

Data of J2 mortality were previously verified for normality and homoskedasticity of variance according to Shapiro-Wilk (W = 0.920; p-value < 0.0001) and to Levene’s test (F = 75.515; p-value < 0.0001) by using the XLSTAT software. Data were subjected to analysis of variance (ANOVA) ANOVA using XLSTAT 2023 1.1 (https://www.xlstat.com/en/)^47^. Data were then analyzed by one-way analysis of variance and comparing means by the Fisher’s Least Significant Difference pairwise procedure (*p* ≤ 0.05), using the PlotIT 3.2 (Scientific Programming Enterprises, Haslett, MI) statistical software. Probit analysis was applied to calculate the LC_50_ values of both *C. zeylanicum and C. aurantium* EOs on the root-knot nematode J2^44^. Relative gene expression values were subjected to one-way analysis of variance as described above.

## Electronic supplementary material

Below is the link to the electronic supplementary material.

Supplementary Material 1

## Data Availability

All data generated or analysed during this study are included in this published article.
