# Supplementary material for: Molecular mechanism of Cinnamomum zeylanicum and Citrus aurantium essential oils against the root-knot nematode, Meloidogyne incognita
Source: Sci Rep. 2025 Feb 19;15:6077. doi: 10.1038/s41598-025-90529-8 (PMC11840038; doi:10.1038/s41598-025-90529-8)

**Supplementary Information**

**Molecular mechanism of** *Cinnamomum zeylanicum* **and** *Citrus aurantium* **essential oils against the root-knot nematode,** *Meloidogyne incognita*

**Supplementary Figure S1:** Percentage mortality (means of four replicates) of infective juveniles of the root-knot nematode *Meloidogyne incognita* exposed for 4, 8 or 24 hours to different concentrations of EOs from *Cinnamomum zeylanicum* and *Citrus aurantium*, and Oxamyl. Within each EO, bars marked with the same letter are not significantly different (p ≤ 0.05) according to the Least Significant Difference Test.


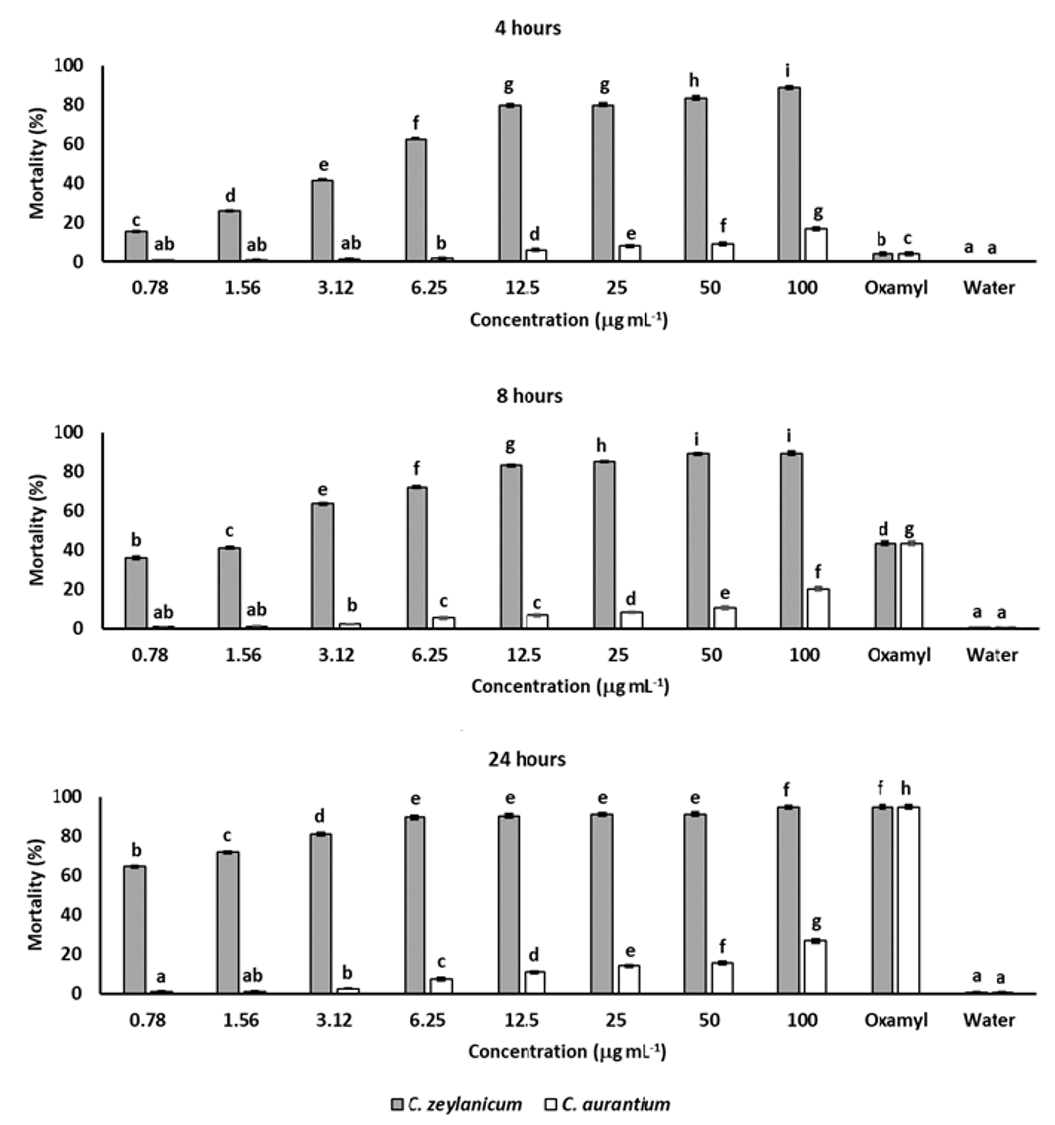

Supplement: Supplementary file 1 — Supplementary Material 1 [file 41598_2025_90529_MOESM1_ESM.docx]
